# Association of Virulence Markers With Resistance to Oral Antibiotics in Escherichia coli Isolates Causing Uncomplicated Community-Acquired Cystitis

**DOI:** 10.7759/cureus.39458

**Published:** 2023-05-24

**Authors:** Shruti Radera, Jyotsna Agarwal, Sugandha Srivastava, Prashant Gupta, Amita Pandey

**Affiliations:** 1 Microbiology, King George's Medical University, Lucknow, IND; 2 Microbiology, Dr. Ram Manohar Lohia Institute of Medical Sciences, Lucknow, IND; 3 Obstetrics and Gynecology, King George's Medical University, Lucknow, IND

**Keywords:** urinary tract infection, escherichia coli, community-acquired cystitis, virulence factors, multidrug resistant, cystitis, lower uti, oral antibiotics

## Abstract

Introduction: Uropathogenic *Escherichia coli *(UPEC) strains equipped with putative virulence factors (VFs) are known to cause approximatel*y* 90% of lower urinary tract infections (UTIs) or cystitis affecting individuals of all age groups. Only limited laboratory-based data on the correlation of antimicrobial resistant patterns and VFs of UPEC are available.

Materials and methods: A total of 100 non-duplicate *E. coli* isolates associated with community-acquired UTIs in sexually active women were analysed for antimicrobial susceptibility patterns and putative virulence-associated genes*.* Antimicrobial susceptibility testing (AST) was carried out by the Kirby-Bauer disk diffusion method, and results were interpreted as per Clinical and Laboratory Standards Institute (CLSI) guidelines. The isolates non-susceptible to ≥1 agent in ≥3 different antimicrobial categories were considered multidrug-resistant (MDR). Multiplex polymerase chain reaction assay was performed on each *E. coli* isolate to characterize putative virulence genes (VGs) such as *papA*, *malX, PAI*, *ibeA*, *fimH*, *fyuA*, *sfa/focDE*, *papGIII*, *iutA*,* papGI*, *kpsMTII*,* hlyA*, *papGII*, *traT*, *afa/draBC*, *cnf1,* *vat*, and *yfcV.*

Results: Capsule synthesis gene *kpsMTII *(59%)was the most predominant VG present, followed by serum resistance-associated transfer protein gene *traT *(58%) and adhesin gene *fimH* (57%); however, adhesin gene *papGI* (2%) was the least present. The prevalence of antimicrobial resistance was relatively high for commonly used oral antimicrobials of UTI treatment, such as trimethoprim-sulfamethoxazole (68%) and fluoroquinolones (63%). The majority of isolates were MDR (78%) and resistant to extended-spectrum cephalosporins (63.5%). Isolates resistant to norfloxacin and trimethoprim-sulfamethoxazole were also resistant to almost all available oral antimicrobials. Isolates resistant to extended-spectrum cephalosporins showed increased resistance to aztreonam and trimethoprim-sulfamethoxazole (84.6% each) and fluoroquinolones (ciprofloxacin and norfloxacin; 81.5% each). Fosfomycin and nitrofurantoin were the most sensitive antimicrobials for all these resistant isolates.

In a multivariate analysis, it was found that MDR isolates were associated with many of the VGs; *fimH *(65.4%) being the most frequent followed by *traT *(64.1%). *traT* (66.2%) and *iutA* (60.3%) were most commonly present in *E. coli* isolates resistant to trimethoprim-sulfamethoxazole, while66.7% norfloxacin-resistant isolates have them. Isolates resistant to extended-spectrum cephalosporins were most commonly associated with *fimH* and *traT* (66.2% each). However, *E. coli* isolates positive for *sfa/focDE *and* vat *were more sensitive to norfloxacin and trimethoprim-sulfamethoxazole and were non-MDR strains predominantly (p < 0.05). Only two VGs (*fimH* and *traT*)* *were significantly associated with MDR strains.

Discussion: The results of the present study clearly show the association of VFs with some of the commonly used oral antibiotics emphasizing the need for further molecular studies and surveillance programs to monitor drug-resistant UPEC so as to form optimized diagnostic stewardship and appropriate regimen for patient treatment. The reason behind this phenomenon of association has not been studied in much detail here but it can be assumed that genes responsible for drug resistance may share neighbouring loci with VGs on the mobile genetic elements (e.g., plasmid), which transfer together from one bacterium to another.

## Introduction

Cystitis or lower urinary tract infection (UTI) is one of the most frequent bacterial infections affecting individuals of all age groups, including both outpatients and inpatients, causing significant morbidity throughout the world [1]. The chances of developing UTI are significantly higher in females than males due to their anatomical structure (short urethra) and hormonal milieu. It is estimated that around 50% of women will develop UTI once in their lifetime. Approximately 90% of UTIs are caused by *Escherichia coli, *also termed uropathogenic *E. coli *(UPEC) strains [1]. These UPEC strains are known to express certain putative virulence factors (VFs), such as adhesins, toxins, and capsules, which help them to invade, establish, and survive in the urinary tract, and prevent their detachment while urinating [2]. It has been observed that the severity of UTIs from being asymptomatic and uncomplicated to complicated infection with sequelae depends upon the subset and frequency of VFs present in UPEC strains, adhesive molecules perhaps being the most important determinants of pathogenicity [3].

The phenomenon of antimicrobial resistance has been a major problem for many years. Treatment for uncomplicated community-acquired UTI is achieved most of the time empirically without waiting for a culture report of urine specimen. Cephalosporins, fluoroquinolones, and trimethoprim-sulfamethoxazole are often used to treat patients with UTIs; but excessive and erratic use of antibiotics has led to the development of multidrug-resistant (MDR) strains, causing these drugs to be ineffective in many cases. The increased frequency of drug resistance/MDR UPEC is related to inadequate antibiotic empirical therapies without any laboratory evidence of antibiotic susceptibility profile, which finally leads to ineffective treatment of UTIs [4]. Only limited laboratory-based data on resistant UPEC causing community-acquired UTIs are available; furthermore, these studies do not include detailed molecular characterization of the isolates [5].

Details of resistant patterns and molecular characterization of UPEC are not frequently available. Hence, reliance only on clinical presentation should be avoided in the presence of limited laboratory data of resistant patterns and molecular characterization of UPEC. Therefore, the detection and identification of drug resistance, along with virulence and virulence-related gene profiles, accommodate a more robust and more accurate characterization for drug-resistant UPEC pathotypes, as the presence of drug resistance among UPEC has led to unsuccessful or prolonged treatment [6].

Resistance to many antimicrobials in bacterial strains is often associated with the transfer of plasmids from one strain to another that may also carry some of the virulence genes with them. The presence of antimicrobial resistance genes can be attributed to DNA mutations or horizontal transfer of drug resistance among UPEC strains [6]. Similar to antimicrobial resistance genes, virulence genes are also located on chromosomes or mobile genetic elements such as plasmids and transposons. Thus, the association between antimicrobial resistance and virulence genes is quite understood [6]. It has been reported in many studies that there is some correlation between resistance patterns and virulence genes, which may help bacteria to survive effectively. An earlier study done by Neamati et al. reported that *traT* was more prevalent in multidrug-resistant *E. coli *and could be considered a potential target for therapeutic intervention [7].

Proper understanding, detection, and identification of antibiotic-resistant patterns and their association with virulence genes can be used to develop better and more targeted regimens for drug-resistant UPEC and prevention of antibiotic misuse. Thus, the present study was planned to study antibiotic susceptibility profile and any correlation of VFs with antibiotic resistance in *E. coli* isolates associated with community-acquired UTIs in sexually active women.

## Materials and methods

A total of 100 non-duplicate *E. coli* isolates associated with community-acquired UTIs in sexually active women attending the OPD of Obstetrics and Gynaecology were studied in the Department of Microbiology at King George's Medical University (KGMU), Lucknow, India. The study protocol was approved by the ethical committee of the host institution (reference no.: 78th ECM II BMD-Ph.D./P1).

Patient enrolment criterion, urine sample processing, and antimicrobial susceptibility testing (AST) were performed as per our previously published paper [8]. AST was carried out using the Kirby-Bauer disk diffusion method, and results were interpreted as per the Clinical and Laboratory Standards Institute (CLSI) guidelines described in the AST interpretation criterion in Table 1 [9]. The isolates non-susceptible to ≥1 agent in ≥3 different antimicrobial categories were considered MDR [10].

**Table 1:** Antimicrobial susceptibility testing (AST) interpretation criterion

Antimicrobial category	Antimicrobial agent (zone criteria for sensitive strains (in millimetres))
Penicillin	Ampicillin (≥17)
Penicillin + β-lactamase inhibitors	Amoxycillin-clavulanic acid (≥18)
First-generation cephalosporins	Cefazolin (≥15)
Third-generation cephalosporins	Ceftazidime (≥21)
	Cefotaxime (≥26)
Quinolones	Norfloxacin (≥17)
	Ciprofloxacin (≥21)
Nitrofurans	Nitrofurantoin (≥17)
Trimethoprim	Trimethoprim-sulfamethoxazole (≥16)
Aminoglycosides	Gentamicin (≥15)
	Amikacin (≥17)
Cephamycin	Cefoxitin (≥18)
	Piperacillin/tazobactam (≥21)
Monobactams	Aztreonam (≥21)
Carbapenems	Meropenem (≥23)
	Imipenem (≥23)

Multiplex polymerase chain reaction assay was performed on each *E. coli* isolate to characterize the putative virulence genes in the following pools, as described by Johnson and Stell (2000) [11]: pool 1: *papA* (P-fimbriae), *malX*, *PAI* (pathogenicity island marker), *ibeA* (invasion of brain endothelium protein A), and *fimH* (type 1 fimbriae); pool 2: *fyuA* (ferric yersiniabactin receptor), *sfa/focDE* (central (consensus) region of the *sfa/foc* operon), *papGIII* (P fimbriae tip adhesin III), and *iutA* (ferric aerobactin receptor); pool 3: *papGI* (P fimbriae tip adhesin I), *kpsMTII* (group II capsular polysaccharide synthesis), and *hlyA* (α-hemolysin); pool 4: *papGII* (P fimbriae tip adhesin II) and *traT* (transfer protein); pool 5: *afa/draBC* (fimbriae/Dr-binding fimbriae) and *cnf1* (cytotoxic necrotising factor 1); pool 6: *vat* (vacuolating autotransporter toxin) and *yfcV* (major subunit of putative fimbriae) [12].

J96 pyelonephritis isolate (JJ079), 2H25 urosepsis isolate (BUTI 3-1-4), V27 urosepsis isolate (BUITI 1-5-1), L31 canine UTI isolate (LOW 31), UTI89 (cystitis isolate), and 2H16 urosepsis isolate (BUTI 3-1-2) were used as positive controls while human faecal isolate JJ055 was used as a negative control, which were kindly provided by Dr. J.R. Johnson, Director, Infectious Diseases Fellowship Program, University of Minnesota, USA.

Statistical analysis

Data analysis was carried out using SPSS version 20.0 statistical software package (IBM Corp., Armonk, NY). The relationship between VFs and antibiotic susceptibility was determined using Pearson's chi-square test or Fisher's exact test. To facilitate the final analysis, the isolates showing intermediate susceptibility were grouped with the sensitive strains. The descriptive statistics for various variables were reported as percentages for qualitative variables and a p-value < 0.05 was considered significant.

## Results

Resistance pattern of antibiotics commonly used in the treatment of uncomplicated community-acquired UTI

Details of the antimicrobial resistance pattern of *E. coli *isolates resistant to antimicrobials commonly used in the treatment of community-acquired UTI are shown in Table 2. Isolates resistant to norfloxacin and trimethoprim-sulfamethoxazole were also resistant to almost all available oral antimicrobials such as ampicillin, cefazolin, and extended-spectrum cephalosporins (Table 2). Isolates resistant to extended-spectrum cephalosporins were most commonly associated with *fimH* and *traT* (66.2% each). Isolates resistant to extended-spectrum cephalosporins showed increased resistance to aztreonam and trimethoprim-sulfamethoxazole (84.6% each) and fluoroquinolones (ciprofloxacin and norfloxacin; 81.5% each). None of the isolates showed resistance to fosfomycin. Apart from fosfomycin, nitrofurantoin was the most sensitive antimicrobial for all these resistant isolates.

**Table 2:** Antimicrobial resistance pattern of E. coli isolates resistant to antimicrobials commonly used in the treatment of community-acquired UTI

Antimicrobial category	Antimicrobial agent	Norfloxacin (n = 63)	Trimethoprim-sulfamethoxazole (n = 68)	Extended-spectrum cephalosporins (n = 65)	Multidrug-resistant (n = 78)
Penicillin	Ampicillin	62 (98.4)	65 (95.6)	64 (98.5)	76 (97.4)
Penicillin + β-lactamase inhibitors	Amoxycillin-clavulanic acid	60 (95.2)	63 (92.6)	62 (95.4)	73 (93.6)
First-generation cephalosporins	Cefazolin	58 (92.1)	61 (89.7)	64 (98.5)	70 (89.7)
Third-generation cephalosporins	Ceftazidime	53 (84.1)	53 (77.9)	-	63 (80.8)
	Cefotaxime	53 (84.1)	54 (79.4)	-	64 (82.1)
Quinolones	Norfloxacin	-	55 (80.9)	53 (81.5)	63 (80.8)
	Ciprofloxacin	61 (96.8)	55 (80.9)	53 (81.5)	63 (80.8)
Nitrofurans	Nitrofurantoin	2 (3.17)	2 (2.9%)	2 (3.07)	2 (2.56)
Trimethoprim	Trimethoprim-sulfamethoxazole	55 (87.3)	-	55 (84.6)	66 (84.6)
Aminoglycosides	Gentamicin	24 (38.1)	22 (32.4)	21 (32.3)	24 (30.8)
	Amikacin	15 (23.8)	13 (19.1)	15 (23.1)	17 (21.8)
Cephamycin	Cefoxitin	12 (19.1)	14 (20.6)	15 (23.1)	15 (19.2)
	Piperacillin/tazobactam	11 (17.5)	9 (13.2)	10 (15.4)	11 (14.1)
Monobactams	Aztreonam	49 (77.8%)	48 (70.6)	55 (84.6)	56 (71.8)
Carbapenems	Meropenem	15 (23.8)	13 (19.1)	10 (15.4)	16 (20.5)
	Imipenem	13 (20.6)	13 (19.1)	14 (21.5)	15 (19.2)

**Figure 1:**
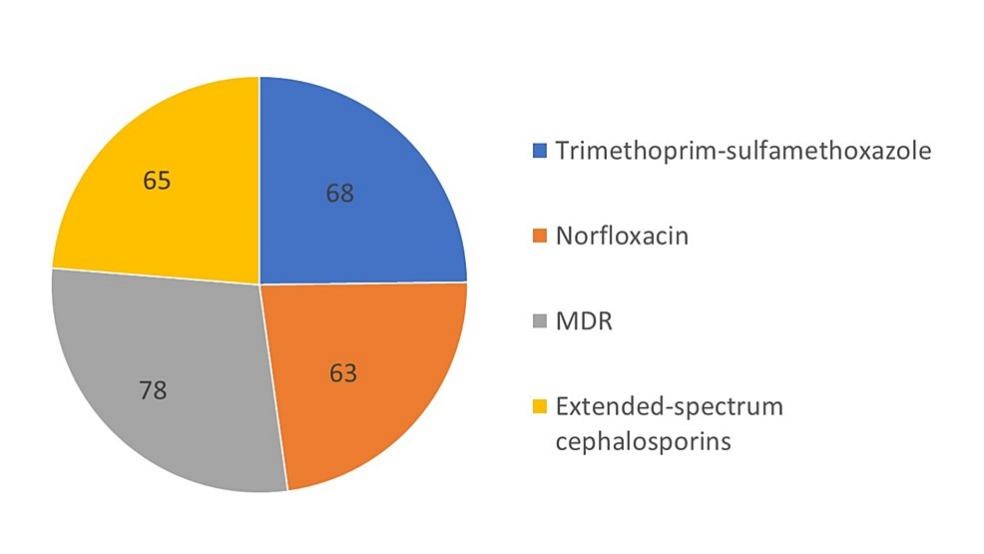
Percentage frequency of resistance to antibiotics commonly used in the treatment of UTIs MDR: multidrug-resistant.

The prevalence of antimicrobial resistance was relatively high for commonly used antimicrobials of UTI treatment, such as trimethoprim-sulfamethoxazole (68%) and fluoroquinolones (63%). The majority of isolates were resistant to extended-spectrum cephalosporins ceftazidime (63%) and cefotaxime (64%) (Figure 1). Among isolates resistant to cefotaxime, two were sensitive to ceftazidime while one isolate was resistant to ceftazidime only and sensitive to cefotaxime. Thus, 65% of *E. coli* isolates were found resistant to extended-spectrum cephalosporins. Multidrug resistance was found in 78% of isolates (Figure 1).

Virulence genotyping

**Figure 2:**
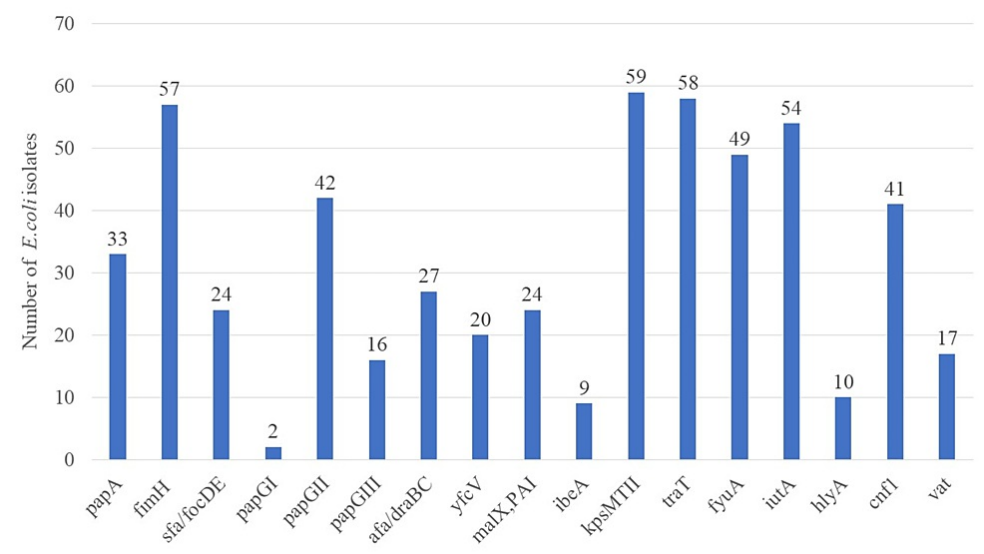
Distribution of virulence factors among E. coli isolates associated with community-acquired UTI

Capsule synthesis gene *kpsMTII* (59%) was the most predominant virulence gene present, followed by serum resistance-associated transfer protein gene *traT* (58%) and adhesin gene *fimH* (57%) (Figure 2). The gene cluster associated with P-fimbrial structural subunits, i.e., *papA*, known to be associated with the formation of P-fimbriae was present in 33% of isolates only whereas adhesin gene *papGI* (2%) was the least frequent gene associated with *E. coli* isolates obtained from the urine of community-acquired uncomplicated UTIs (Figure 2).

Co-relation of virulence factors with antibiotic sensitivity pattern

Any statistical association between antibiotic sensitivity patterns and virulence genes of isolates was subsequently investigated. Table 3 shows the prevalence of each virulence gene among isolates resistant to antimicrobials commonly used in the treatment of community-acquired UTIs. *traT* (66.2%) and *iutA* (60.3%) were most commonly present in *E. coli* isolates resistant to trimethoprim-sulfamethoxazole, while iron acquisition gene *iutA* and serum resistance-associated gene *traT* were present in 66.7% of norfloxacin-resistant isolates. Isolates resistant to extended-spectrum cephalosporins were most commonly associated with *fimH* and *traT* (66.2% each).

**Table 3:** Distribution of virulence genes and their association with antibiotic resistance and multidrug-resistant isolates P-values shown in bold have a significant association with resistant strains whereas italic p-values have a significant association with sensitive strains. MDR: multidrug-resistant.

Virulence genes	MDR (n = 78)		Trimethoprim-sulfamethoxazole (n = 68)		Norfloxacin (n = 63)		Extended-spectrum cephalosporins (n = 65)	
	Number	p-value	Number	p-value	Number	p-value	Number	p-value
papA	29	0.094	24	0.477	23	0.330	24	0.256
fimH	51	0.001	40	0.591	38	0.382	43	0.012
sfa/focDE	14	0.008	10	0.002	11	0.046	12	0.077
papGI	1	0.393	1	0.540	1	1.00	1	1.00
papGII	37	0.038	30	0.532	29	0.286	34	0.004
papGIII	14	0.512	11	0.944	10	0.964	12	0.360
afa/draBC	22	0.609	20	0.428	17	0.996	20	0.247
yfcV	18	0.228	16	0.198	13	0.836	15	0.295
malX, PAI	22	0.064	17	0.733	19	0.060	16	0.844
ibeA	6	0.408	7	0.715	5	0.722	5	0.716
kpsMTII	47	0.631	40	0.958	41	0.107	37	0.565
traT	50	0.020	45	0.016	42	0.022	43	0.024
fyuA	42	0.068	36	0.250	34	0.195	36	0.082
iutA	45	0.163	41	0.066	42	0.001	37	0.424
hlyA	9	0.452	7	1.000	8	0.315	9	0.159
cnf1	31	0.631	23	0.033	23	0.233	28	0.565
vat	7	<0.001	8	0.042	5	0.002	6	0.005

On further analysis, we found that few virulence genes showed significant association with isolates resistant to various antibiotics (Tables 3-5). The gene cluster associated with P-fimbrial structural subunits, i.e., *papA*, *papGI*, and *papGIII*, and toxin-producing gene *hlyA* were equally distributed in both resistant as well as sensitive *E. coli* isolates (Table 3). However, *E. coli* isolates positive for *sfa/focDE* and *vat* were more sensitive to norfloxacin and trimethoprim-sulfamethoxazole and were non-MDR strains predominantly (p < 0.05), i.e., these genes were more associated with sensitive strains.

**Table 4:** Significant association of virulence genes with multidrug-resistant E. coli isolates and resistance to trimethoprim-sulfamethoxazole P-values shown in bold have a significant association with resistant strains whereas italic p-values have a significant association with sensitive strains.

Virulence genes	Total	MDR			Trimethoprim-sulfamethoxazole		
		Yes (n = 78), number (%)	No (n = 22), number (%)	p-value	Resistant (n = 68), number (%)	Sensitive (n = 32), number (%)	p-value
fimH	57	51 (65.4%)	6 (27.3%)	0.001	-	-	-
sfa/focDE	24	14 (17.9%)	10 (45.5%)	0.008	10 (14.7%)	14 (43.8%)	0.002
papGII	42	37 (47.4%)	5 (22.7%)	0.038	-	-	-
traT	58	50 (64.1%)	8 (36.4%)	0.020	45 (66.2%)	13 (19.11%)	0.016
cnf1	41	-	-	-	23 (33.8%)	18 (56.3%)	0.033
vat	17	7 (8.9%)	10 (45.5%)	0.000	8 (11.8%)	9 (28.1%)	0.042

**Table 5:** Significant association of virulence genes with E. coli isolates resistant to norfloxacin and among extended-spectrum beta-lactamase producers P-values shown in bold have a significant association with resistant strains whereas italic p-values have a significant association with sensitive strains.

Virulence genes	Total	Norfloxacin			Extended-spectrum cephalosporins (n = 65)		
		Resistant (n = 63), number (%)	Sensitive (n = 37), number (%)	p-value	Resistant (n = 65), number (%)	Sensitive (n = 35), number (%)	p-value
fimH	57	-	-	-	43 (66.2%)	14 (40%)	0.012
sfa/focDE	24	11 (17.5%)	13 (35.1%)	0.046	-	-	-
papGII	42	-	-	-	34 (52.3%)	8 (22.9%)	0.004
traT	58	42 (66.7%)	16 (43.2%)	0.022	43 (66.2%)	15 (42.9%)	0.024
iutA	54	42 (66.7%)	12 (32.4%)	0.001	-	-	-
vat	17	5 (7.9%)	12 (32.4%)	0.002	6 (9.2%)	11 (31.4%)	0.005

**Figure 3:**
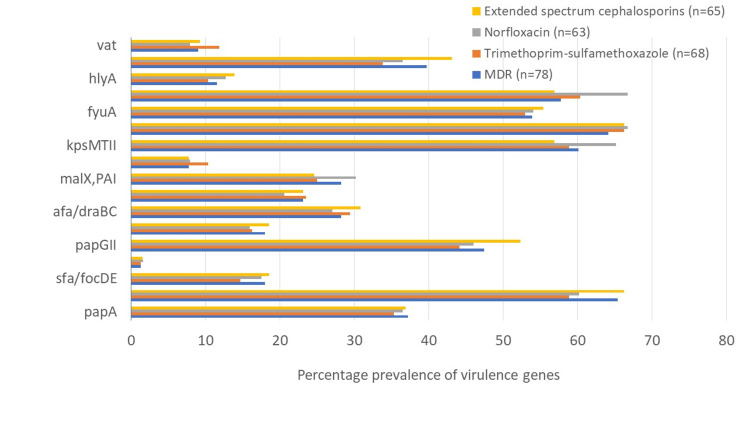
Distribution of virulence genes according to antibiotic resistance profile among E. coli isolates MDR: multidrug-resistant.

In a multivariate analysis to find the correlation between the virulence genes and antimicrobial resistance, it was found that MDR isolates were associated with many of the virulence genes, fimbrial gene *fimH* (65.4%) being the most frequent, followed by serum resistance *traT* (64.1%) (Table 4 and Figure 3).

*sfa/focDE* was present in 17.9% of MDR strains and 45.5% of non-MDR strains while *vat* was present in 8.9% and 45.5% of MDR and non-MDR strains, respectively (Table 4). Only two virulence genes such as *fimH* and *traT* were significantly associated with MDR strains. Among trimethoprim-sulfamethoxazole-resistant isolates, only *traT* was found significantly associated while *sfa/focDE*, *cnf1*, and *vat* showed significant association with trimethoprim-sulfamethoxazole-sensitive isolates.

Among norfloxacin-resistance isolates, only *traT* and *iutA* were found significantly associated (p < 0.05), whereas *fimH*, *papGII*, and *traT* were significantly associated with isolates resistant to extended-spectrum cephalosporins (p < 0.05; Table 5).

## Discussion

*E. coli* inhabits the gastrointestinal tract of humans in a symbiotic relationship, which helps in maintaining normal intestinal homeostasis by promoting the stability of intestinal microbial flora [13]. However, if the host is immunocompromised or gastrointestinal barriers are breached, even non-pathogenic strains of* E. coli *can cause diseases [14]. It is known that strains of *E. coli* causing extraintestinal disease originate from the normal intestinal flora, diverge from their ecological niche, and cause infection after acquiring some unique VFs [3]. These diverged *E. coli* strains obtain various VFs via DNA horizontal transfer by transposons, plasmids, bacteriophages, and pathogenicity islands, resulting in enhanced pathogenic potential [3,15]. UPEC are a special subset of faecal *E. coli, *which can enter and colonize the urinary tract and cause infection.

The steady increase in antibiotic resistance is being reported in many UPEC strains [4,16]. β-lactams, trimethoprim-sulfamethoxazole (TMP/SMX), fluoroquinolones, and nitrofurantoin are the most common antibiotics used in the treatment of community-acquired UTIs [17]. Improper stewardship, inappropriate use of unprescribed antibiotics, and over-prescription of broad-spectrum antibiotics are among a few contributing factors to the rapid emergence of antibiotic resistance. The presence of MDR is interconnected with high rates of imperfect empirical antibiotic therapy, which ultimately leads to treatment failure in patients suffering from UTI [4,16,18]. Therefore, in recent years, the treatment of community-acquired UTIs is becoming a global concern due to the emergence of MDR *E. coli* [16].

Although antibiotic resistance genes and virulence genes are believed to be developed in different timescales, there are chances of the interplay between virulence genes and antibiotic resistance genes under selection pressure [19]. It was believed that strains with antibiotic resistance might be coupled with fewer virulence genes, but this may not always be accurate. Many published data revealed the relationship between resistance and virulence is adjusted in such a way that it is beneficial for pathogen survival [20]. VFs are essential for the bacteria to overcome the host defence system, colonize, and survive, while the acquisition of antibiotic resistance helps bacteria to overcome antimicrobial therapies and to adapt to colonize adverse environments [19,21]. Thus, establishing any correlation between virulence and antibiotic resistance can further help in studying targeted/alternative drug therapy [16,22]. This study was planned to determine the presence of virulence genes among *E. coli* isolates and their correlation with antimicrobials commonly used in the treatment associated with community-acquired UTI.

Capsule synthesis gene *kpsMTII* (59%) was the most predominant virulence gene present, followed by serum resistance-associated transfer protein gene *traT* (58%) and adhesin gene *fimH* (57%). The gene cluster associated with P-fimbrial structural subunits, i.e., *papA*, known to be associated with the formation of P-fimbria, was present in 33% of isolates. Distribution of adhesins *papGI*, *papGII*, and papGIII was 2%, 42%, and 16%, respectively, which was quite like earlier reported observation by Kudinha et al. (2012) [23]. Interestingly, the distribution of adhesins *afa*/*draBC* was found, which contrasts with previously published studies reporting its low prevalence [24].

Earlier studies have reported the estimated prevalence of resistance in high-income countries as 53.4% for trimethoprim and 2.1% for ciprofloxacin [16]. In comparison, low and middle-income countries showed higher resistance rates for ciprofloxacin (26.8%) [16]. We found similar results as observed earlier, demonstrating relatively higher resistance to commonly used antimicrobials for the treatment of UTIs, such as trimethoprim-sulfamethoxazole (68%) and fluoroquinolones (63%).

The majority of the study isolates were resistant to extended-spectrum cephalosporins, ceftazidime (63%), and cefotaxime (64%); however, overall resistance to extended cephalosporins was found among 65% of isolates, as among isolates resistant to cefotaxime, two were sensitive to ceftazidime while one isolate was resistant to ceftazidime only and sensitive to cefotaxime. Such a higher degree of resistance among extended-spectrum cephalosporins can be attributed to inappropriate prescription by physicians, negligible toxicity and wide spectrum of oral drugs, and over-the-counter availability of antibiotics [16]. Indeed, it has also been noted that isolates resistant to extended-spectrum cephalosporins themselves show more resistance to aminoglycosides, ciprofloxacin, and trimethoprim-sulfamethoxazole, which may be due to sharing of resistance genes on the same plasmid [25].

About 78% of *E. coli *isolates were MDR in the present study. Gatya et al. (2022) reported around 100% MDR strains among outpatients in their study [26]. High rates of resistance to various antibiotics among UPEC have been reported in many previous studies [27,28]. Such a high resistance can be explained due to the fact that *E. coli* has developed resistance to almost every class of antimicrobials introduced to treat human and animal infections [26].

Various theories have been proposed for such a high resistance apart from misuse or inappropriate prescription of an antibiotic, such as the irrational introduction of antibiotics in the food chain. Several studies have shown that multi-drug resistance is easily transferable from one ecosystem to another via direct or indirect contact with contaminated animal or their product, the environment, contaminated soil, or water [25]. Irrational use of antibiotics among animals to increase production or prophylactic use of antibiotics among them to prevent them from getting an infection or their use in crop culture is also responsible for the spread of antibiotic resistance [25]. Thus, the emergence of antibiotic resistance in the food chain has emerged as a global health concern particularly due to the emergence of carbapenem-resistant strains or strains having co-resistant genes for many antibiotics. These strains have the capability of high genetic exchange and are a serious threat to the world as random genetic exchange could lead to the development of new and more resistant and with higher virulence potential unknown to the human immune system. These antibiotic-resistant strains can enter the human ecosystem either through direct contact, e.g., with animal handlers and their family members, or through the food chain [25].

Mobile genetic elements like integrons are competent to attain new genes by recombination process, which leads to incorporation and subsequent expression of new genetic material in bacteria. This phenomenon of gene acquisition is responsible not only for bacterial evolution by enabling bacteria to adapt to the changing surrounding environment but also plays an important role in acquiring drug resistance [29]. Genes responsible for drug resistance and phylogroups can share neighbouring loci and may be transferred from one bacterium to another [29].

Proper understanding, detection, and identification of antibiotic-resistant pattern and their association with virulence genes can be used to develop stronger and appropriate regimens for drug-resistant UPEC and prevent antibiotic misuse. The findings of the current study demonstrated the association of several VFs with resistance to one or more antibiotics in some isolates. We found that isolates with reduced susceptibility to trimethoprim-sulfamethoxazole were more frequently associated with *traT* (45/68), followed by *iutA* (41/68) and *kpsMTII* (40/68), whereas extended-spectrum cephalosporins isolates were more regularly associated with *fimH* and *traT* (43/65 each).

We observed that several MDR isolates have increased frequency of *fimH*, *papGII*, *kpsMTII*, *traT*, *fyuA*, etc.; however, only *fimH*, *papGII*, and serum resistance gene *traT* were significantly associated with MDR isolates while *sfa/focDE* adhesin and toxin gene *vat* were associated considerably with non-MDR trimethoprim-sulfamethoxazole-sensitive and norfloxacin-sensitive strains.

Ochoa et al. (2016) have observed that many MDR-UPEC isolates have a high association for *fimH* and toxin gene *hlyA* [30]. Serum resistance gene was more significantly associated with multi-drug resistance, including trimethoprim-sulfamethoxazole and norfloxacin-resistance isolates (p < 0.05). The gene responsible for iron acquisition (*iutA*) was significantly distributed among norfloxacin-resistant strains. Neamati et al. (2015) also reported that *traT* was more prevalent in MDR *E. coli* and could be considered a potential target for therapeutic intervention [7]. Our results are in agreement with previous studies, which emphasized that increased virulence may either be related to antibiotic-resistant or sensitive strains [19].

Though the reason behind this phenomenon of association has not been studied in much detail here, it can be assumed that genes responsible for drug resistance may share neighbouring loci with virulence genes on the mobile genetic elements (e.g., plasmid). When a transfer takes place in bacteria, drug resistance-causing genes may carry virulence genes along with them from one bacterium to another [19,24]. Simultaneously, gene transfer events (e.g., conjugation and transduction) and a large genetic library will contribute to bacterial strains to compensate for or overcome fitness costs, resulting in the successful colonization and emergence of resistant as well as virulent strains [19].

Limitations of the present study include the detection of antibiotic resistance using the disk diffusion method instead of a more precise minimum inhibitory concentration detection method and the study population of younger women presenting in an outpatient setting to a tertiary care hospital, which may not be a true representative of the community at large.

## Conclusions

The increasing emergence of antibiotic resistance and its association with virulence genes is indicated in the findings of the present study. Though the reason behind this phenomenon of association has not been studied in much detail here, it can be assumed that genes responsible for drug resistance may share neighbouring loci with virulence genes on the mobile genetic elements (e.g., plasmid). When a transfer takes place in bacteria, drug resistance-causing genes may carry virulence genes along with them from one bacterium to another. Future in-depth investigations would provide broader insights into the association and co-selection dynamics of antimicrobial resistance among UPEC isolates, which can further be explored for comprehensive research studies emphasizing upon new therapeutic medicines and vaccines against these putative virulence factors. Upcoming research must continue to explore the latest changes in the epidemiology of UPEC isolates to assist in timely intervention for patient treatment and prevention of antibiotic misuse and the development of optimized diagnostic stewardship.
